# Glymphatic Dysfunction Mediates the Influence of White Matter Hyperintensities on Episodic Memory in Cerebral Small Vessel Disease

**DOI:** 10.3390/brainsci12121611

**Published:** 2022-11-24

**Authors:** Zhihong Ke, Yuting Mo, Jiangnan Li, Dan Yang, Lili Huang, Zhiyuan Yang, Ruomeng Qin, Chenglu Mao, Weiping Lv, Yanan Huang, Zheqi Hu, Bing Zhang, Yun Xu

**Affiliations:** 1 Department of Neurology, Nanjing Drum Tower Hospital Clinical College of Nanjing Medical University, Nanjing 210008, China; 2 Department of Neurology, Drum Tower Hospital, Medical School and The State Key Laboratory of Pharmaceutical Biotechnology, Institute of Brain Science, Nanjing University, Nanjing 210008, China; 3 Department of Radiology, The Affiliated Drum Tower Hospital of Nanjing University Medical School, Nanjing University, Nanjing 210008, China; 4 Jiangsu Key Laboratory for Molecular Medicine, Medical School of Nanjing University, Nanjing 210008, China; 5 Jiangsu Province Stroke Center for Diagnosis and Therapy, Nanjing 210008, China; 6 Nanjing Neurology Clinic Medical Center, Nanjing 210008, China

**Keywords:** glymphatic system, cerebral small vessel disease, cognitive impairment, white matter hyperintensities

## Abstract

Glymphatic dysfunction has been linked to cognitive decline in several neurodegenerative diseases. In cerebral small vessel disease (CSVD), the mechanism of white matter hyperintensities (WMH)-related cognitive impairment (CI) is still under investigation. The diffusion tensor image (DTI) analysis along the perivascular space (ALPS) method has been considered to be a reliable parameter to evaluate glymphatic function. Therefore, we applied the ALPS-index to determine the influence of glymphatic function on CI in CSVD. In total, 137 CSVD patients (normal cognitive group, mild CI group, and dementia group) and 52 normal controls were included in this study. The ALPS-index was calculated based on the DTI. Correlation analyses and mediation analysis were conducted to examine the relationship between glymphatic function and cognition. Remarkable differences in the ALPS-index were observed between subjects with and without CI. The ALPS-index was negatively correlated with age, WMH volume, and general cognitive function in all CSVD patients. In the mild CI group, the ALPS-index was independently positively related to episodic memory, and mediated the relationship between WMH volume and episodic memory. In conclusion, the ALPS-index is a potential marker for early recognition of CI in CSVD. Glymphatic dysfunction mediates the relationship between WMH and CI in CSVD.

## 1. Introduction

Cerebral small vessel disease (CSVD) is a clinical syndrome associated with damaged cerebral small vessels, which manifests as mood disorders, abnormal gait, stroke, and cognitive decline [1,2]. White matter (WM) hyperintensities (WMH), lacunar infarction (LI), cerebral microbleeds (CMBs), enlarged perivascular space (EPVS), and brain atrophy are common imaging features of CSVD [2]. CSVD impacts almost everyone over the age of ninety, and has become a leading cause of cognitive decline, accounting for roughly 45% of dementia cases in the elderly [3]. Due to the refractory character of dementia, scholars have been committed to the early recognition of and intervention for cognitive impairment (CI) [4]. However, studies on reliable indicators for prediction and early intervention of CI are still underway.

The glymphatic system (GS), which was first characterized in 2012, is a novel excretory pathway for metabolic waste in the central nervous system [5]. The GS reportedly transports cerebrospinal fluid (CSF) from the perivascular space (PVS) of arterioles to the neurointerstitium via aquaporin-4 (AQP4) at the astrocytic endfeet. Here, CSF mixes with the interstitial fluid (ISF), allowing waste products to be excreted from the brain through the perivenous space [6]. Recently, studies have proposed that dysfunction of the GS may contribute to CI in neurodegenerative diseases [7].

The GS may be closely correlated to vasculopathy [8]. Indeed, several vascular risk factors, including diabetic mellitus and hypertension, have been shown to be related to glymphatic function [9]. Astroglia, a key cellular component in the neurovascular unit, is involved in transport in the GS [10]. The PVS, which is often enlarged in CSVD, is an important anatomical channel for the GS to enable ISF circulation [10]. Moreover, pathological changes in cerebral small vessels in CSVD can affect cerebral arterial pulsatility and venous reflux, which participate in the cycle efficiency of the GS [11]. These reports indicate that the GS may be dysfunctional in CSVD. However, little is known about the effects of glymphatic function on cognition in CSVD.

To date, two methods have been used to assess glymphatic function. First, magnetic resonance imaging (MRI) after intrathecal injection of gadolinium-based contrast agent (GBCA), known as glymphatic-MRI, could intuitively reflect the function of the GS [12]. However, intrathecal injection of GBCA is an invasive procedure with low patient tolerance. Furthermore, gadolinium may be deposited in the central nervous system and cause damage. Taoka et al. [13] defined a new parameter, termed the index of diffusion function along the perivascular space (ALPS-index), which can be obtained non-invasively from diffusion tensor images (DTI)/MRI. The capability of the ALPS-index to measure the function of the GS has been confirmed by classical glymphatic-MRI [14].

The ALPS-index has previously shown a non-negligible correlation with cognitive function in neurodegenerative diseases, and may, therefore, have the potential to predict the occurrence of dementia, such as in Alzheimer’s Disease (AD) and Parkinson’s Disease (PD) [13,15,16]. Here, we applied the ALPS-index to CSVD in order to: (1) detect early changes in CI in CSVD using the ALPS-index and differences among groups; and (2) examine how changes in the GS may affect cognitive function in CSVD.

## 2. Methods

### 2.1. Participants

**Figure 1:**
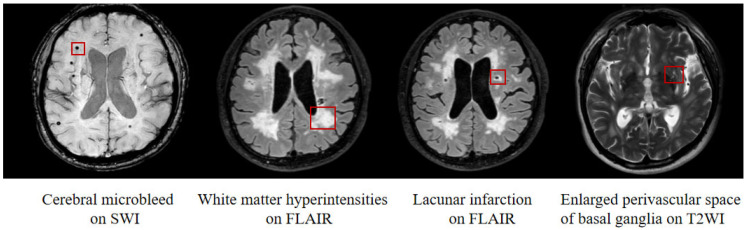
Neuroimaging features of cerebral small vessel disease. From left to right, the images show cerebral microbleed on SWI, white matter hyperintensities on FLAIR, lacunar infarction on FLAIR, and enlarged perivascular space of basal ganglia on T2WI. Abbreviations: SWI: susceptibility weighted imaging; FLAIR: fluid-attenuated inversion recovery imaging; T2WI: T2-weighted imaging.

A total of 137 CSVD patients and 52 normal controls (NC group) were recruited for this study from the Affiliated Drum Tower Hospital of Nanjing University Medical School. All subjects underwent neuropsychological tests and multimodal MRI scans, and background information was collected by an experienced neurologist. CSVD was defined as the presence of symptoms that included CI, motor disturbance, and mood disorders, as well as visualization of the following lesions on MRI: moderate-to-severe WMH (Fazekas score 2–3) and/or LIs (diameter less than 15 mm), with or without EPVS, CMBs (diameter less than 5 mm), and brain atrophy [17]. In order to calculate the ALPS-index, we focused on the EPVS in the basal ganglia (EPVS-bg) (Figure 1). The inclusion criteria of CSVD patients were: (1) right-handedness; (2) age between 45 to 85 years; and (3) a defined CSVD diagnosis by two independent, experienced neurologists. The exclusion criteria included: (1) acute cerebral ischemic stroke, previous cerebral infarctions (diameter larger than 15 mm), or cerebral hemorrhage; (2) other neurological disorders, such as AD, PD, epilepsy, or head trauma; (3) other causes of WMH, such as demyelination, metabolic diseases, immunological diseases, intoxication, or infection; (4) other systemic diseases, such as tumors, hyperthyroidism, hypothyroidism, or systemic lupus erythematosus; and (5) contraindications to the MRI scan.

Based on neuropsychological tests, CSVD patients were divided into three groups: normal cognitive group (CSVD-non-CI group, 56 subjects), mild cognitive impairment group (CSVD-MCI group, 52 subjects), and dementia group (CSVD-VaD group, 29 subjects). The Mini-Mental State Examination (MMSE) [18] was used to identify dementia, and the Montreal Cognitive Assessment (MoCA) (Beijing version) [19] was applied to the diagnosis of MCI. The education-adjusted cut-off values of dementia for MMSE were ≤17 for illiteracy, ≤20 for 1–6 years of education, and ≤24 for >6 years of education. The education-adjusted cut-off values of normal cognitive function for MoCA were >13 for illiteracy, >19 for 1–6 years of education, and >24 for 7–12 years of education [19].

The inclusion criteria of the CSVD-non-CI group were: (1) normal education-adjusted MMSE and MoCA scores; and (2) Clinical Dementia Rating (CDR) score = 0, and Activities of Daily Living (ADL) score = 8. The inclusion criteria for the CSVD-MCI participants were: (1) reporting subjective cognitive decline for six months or more; (2) MoCA score below its cut-off value and MMSE score above its cut-off value adjusted for education level; (3) CDR score = 0.5 and ADL score = 8; and (4) consistent with the AHA/ASA statement (2011) on MCI [20]. The inclusion criteria of the CSVD-VaD group were: (1) an MMSE score associated with dementia; (2) meeting the diagnostic criteria of the AHA/ASA statement (2011) on VaD [20]; and (3) CDR score ≥ 1. Finally, the inclusion criteria of the NC group were: (1) right-handedness; (2) age between 45 to 85 years; (3) normal education-adjusted MMSE and MoCA scores; and (4) rejecting the diagnosis of CSVD. The exclusion criteria of the NC group were the same as the CSVD patients.

This study was approved by the Ethics Committee of the Affiliated Drum Tower Hospital of Nanjing University Medical School (Ethical Approval Code: 2017-079-04). Written informed consent was obtained from all participants.

### 2.2. MRI Scanning

All MRI scans were performed using a Philips 3.0T scanner (Ingenia 3.0T, Philips Medical Systems, Eindhoven, The Netherlands) equipped with a 32-channel head coil. Patients wore noise-canceling headphones to reduce scanner noise and limit head movement. Acquisition parameters for the high-resolution T1-weighted turbo gradient echo sequence were: repetition time (TR) = 8.2 ms, echo time (TE) = 3.8 ms, flip angle (FA) = 8°, number of slices = 192, field of view (FOV) = 256 × 256 mm^2^, acquisition matrix = 256 × 256, and slice thickness = 1.0 mm. Acquisition parameters for the fluid-attenuated inversion recovery (FLAIR) sequence were: TR/TE/inversion time (TI) = 4500/333/1600 ms, number of slices = 200, voxel size = 0.95 × 0.95 × 0.95 mm^3^, and acquisition matrix = 270 × 260. Acquisition parameters for the T2-weighted sequence were: repetition time (TR) = 2750 ms, echo time (TE) = 80 ms, number of slices = 22, acquisition matrix = 230 × 185, and slice thickness = 5 mm. The acquisition parameters of the susceptibility-weighted image sequence were: TR/TE1/deltaTI = 31/7.2/6.2, number of slices = 100, voxel size = 0.6 × 0.9 × 2.4 mm^3^, and acquisition matrix = 230 × 185. The acquisition parameters for the diffusion tensor image (DTI) were: TR = 8565 ms, TE = 71 ms, FOV = 224 × 224 mm^2^, matrix = 112 × 112, slice thickness = 2.5 mm, voxel size = 2 × 2 × 2.5 mm^3^, and acquisition = 32 with diffusion encoding (b = 1000 s/mm^2^) and without diffusion encoding (b = 0 s/mm^2^).

### 2.3. MRI Analysis

#### 2.3.1. DTI-ALPS Analysis

The FMRIB software library (FSL version 5.0.9, https://www.FMRIB.ox.ac.uk/FSL/, accessed on 6 March 2022) was used to pre-process the raw DTI. First, non-brain tissues were removed. Second, eddy current distortions and head motion were corrected using an affine image registration and eddy current program (a toolbox in FSL software). Third, the whole brain diffusion index of each voxel was calculated using the dtifit program (a toolbox in FSL software), and FA and diffusivity values of Dx, Dy, and Dz were generated along the *x*-axis, *y*-axis, and *z*-axis, respectively.

**Figure 2:**
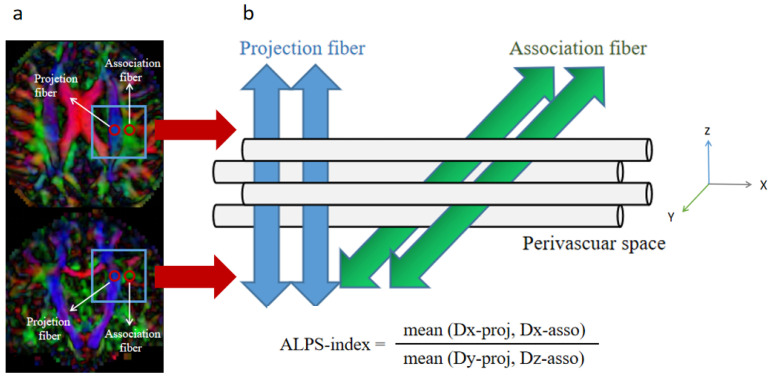
The calculation method used to determine the ALPS-index. (**a**) Two regions of interest were placed on the projection fiber and association fiber using the color FA map. (**b**) Schematic diagram showing the relationship between the orientation of the perivascular space and fibers, and the formula for calculating the ALPS-index. Abbreviation: ALPS-index, the index of Diffusion Tensor Image Analysis along the Perivascular Space.

The ALPS-index was calculated as described previously [13]. In the paraventricular horizontal position, the PVS was horizontal from left to right, and perpendicular to the lateral ventricular wall. In this position, the projection fibers of the cortex were in the craniocaudal direction, and the association fibers (superior longitudinal fasciculus) were anteroposterior. The three structures near the lateral ventricle were perpendicular to each other. Since the fiber bundles in this region were not parallel to the PVS, the diffusivity along the PVS could be independently analyzed in this region. Based on this structure, a spherical ROI containing 12 voxels was placed in the blue area (projection fiber) of the color FA diagram (Figure 2). The average diffusivities along the *x*-axis and *y*-axis were calculated and recorded as Dx-proj and Dy-proj, respectively. A spherical ROI containing 12 voxels was also placed in the green area (association fiber) parallel to the ROI on the projection fiber (Figure 2), and the average diffusivities along the *x*-axis and *z*-axis in the ROI were calculated and recorded as Dx-asso and Dz-asso, respectively. In the projection fiber area, the fibers were shaped along the *z*-axis, while the *x*-axis and *y*-axis were perpendicular to the fiber direction. In the association fiber area, the fibers were oriented along the *y*-axis, while both the *x*-axis and *z*-axis were perpendicular to the fiber direction. The PVS of these two regions was shaped along the *x*-axis, so that Dx-proj and Dx-asso could represent the diffusivity along the PVS without the interference of nerve fibers. Since paraventricular WMHs can increase the diffusivity level in all directions as a whole, this effect was eliminated by using the following formula to calculate the ALPS-index:

ALPS-index = (mean (Dx-proj, Dx-asso))/(mean (Dy-proj, Dz-asso))

3Dslicer software (3Dslicer 4.1.1, https://www.slicer.org/, accessed on 6 March 2022) was used to calculate the diffusivity value. This software automatically stacked the value layers and calculated the average value in two ROIs. Values for Dx-proj, Dx-asso, Dy-proj, and Dz-asso in the ROIs were used to calculate the ALPS-index. In order to eliminate bias, ROIs were drawn by an experienced radiologist who was blinded to the subjects’ information. 

#### 2.3.2. Imaging Biomarkers of CSVD

LST, a toolbox in SPM12 (University College London, Wellcome Trust Centre for Neuroimaging, London, UK), was used to quantify the WMH volume based on the FLAIR sequence [21]. The voxel-based morphometry (VBM) function in SPM8 was used to calculate the global gray matter volume, white matter volume, and intracranial CSF volume from high-resolution T1-weighted turbo gradient echo sequences. The number of LIs and CMBs, as well as the presence or absence of EPVS-bg, were assessed by two experienced radiologists who were blinded to the subjects’ information.

#### 2.3.3. Neuropsychological Measurements

The neuropsychological evaluation of each subject was completed by a neuropsychologist 1 day after receiving the MRI scan. CDR and ADL scores were used to assess the general daily functions of the subjects. MMSE and MoCA scores were used to determine the general cognitive function of the subjects. Episodic memory was assessed using the visual reproduction–long delayed recall (VR–DR) portion of the Wechsler Memory Scale (WMS-VR-DR) and the Huashan version of the Auditory Verbal Learning Test–delayed recall (AVLT–DR). Linguistic function was assessed using the Boston Naming Test (BNT) and category language fluency (CVF). Information processing speed was evaluated using the Trail Making Test A (TMT-A) and Stroop Color and Word Test B (SCWT-B). Executive function was assessed by the Trail Making Test B (TMT-B) and Stroop Color and Word Test C (SCWT-C) [22]. Visuospatial function was evaluated using the Clock Drawing Test (CDT) and Visual Reproduction Test (VRT). It is worth mentioning that the original scores of the TMT and SCWT were based on the completion time and, as a result, were inversely transformed to remain consistent with other cognitive scales. The original scores of all test scales were Z-transformed, and the scores for each cognitive domain were calculated by taking the average from the respective Z-transformed test scale scores [22].

### 2.4. Statistical Analysis

All statistical analyses were performed using SPSS 24.0 software (Chicago, IL, USA). First, continuous variables were tested for normality. Variables that conformed to a normal distribution were expressed as mean ± standard deviation (mean ± s.d.). Variables that did not conform to the normal distribution were represented by the median (interquartile range). Categorical variables were expressed as frequencies and percentages. Continuous variables that conformed to normal distribution were compared using one-way analysis of variance (ANOVA) and the general linear model (GLM). Continuous variables that were not normally distributed were compared using the Kruskal–Wallis test. Chi-square test and Fisher’s exact test were used to compare categorical variables. Bonferroni corrections were performed in post-hoc analyses in ANOVA and GLM.

The number of LIs and CMBs, as well as WMH volume, in the participants did not conform to the normal distribution. In order to maintain data consistency, we performed logarithmic transformation on the WMH volume, number of LIs, and number of CMBs. Next, 1 and 0 were used to represent the presence or absence of EPVS-bg. Pearson correlation and partial correlation were applied in order to examine the relationship between the ALPS-index, CSVD neuroimaging features, and cognitive function. *p* < 0.05 was considered to be statistically significant.

In addition, mediation analysis was performed to determine whether ALPS was involved in the relationship between WMH volume and cognitive performance, adjusted for age, gender, education years, and hypertension. The primary estimates of interest were the degree of changes in the direct path between WMH volume and cognition, labeled c in the bivariate models and c’ in the full mediation models, and the indirect path from WMH volume to cognition through ALPS, which was the product of paths a and b. We computed the bias and corrected 95% confidence intervals for the size of the mediating effects with bootstrapping (k = 5000 samples). The mediating effect was considered to be present if the 95% confidence interval did not contain zero. Mediation analyses were conducted in PROCESS for the SPSS 24.0 framework.

## 3. Results

### 3.1. Demographics and Cognitive Function

Demographically, no significant differences were observed in gender (*p* = 0.991) between the NC, CSVD-non-CI, CSVD-MCI, and CSVD-VaD groups. However, subjects in the NC group were younger than those in the CSVD-non-CI, CSVD-MCI, and CSVD-VaD groups. The education levels were lower in the CSVD-non-CI, CSVD-MCI, and CSVD-VaD groups than in the NC group. With respect to vascular risk factors, only hypertension was found to be significantly different among the four groups (*p* = 0.004). Remarkable differences were observed among all the cognitive parameters in the four groups. Detailed information about the patients is shown in Table 1 and Table 2.

### 3.2. CSVD Neuroimaging Features

Significant differences in the WMH volume, LI numbers, CMB numbers, and prevalence of EPVS-bg were observed between the NC, CSVD-non-CI, CSVD-MCI, and CSVD-VaD groups. Stepwise increases in the neuroimaging features accompanied the development of cognitive decline among the four groups. Notably, no markable differences in global gray matter volume, global white matter volume, or CSF volume were observed in any of the groups. Details about the CSVD neuroimaging features are shown in Table 3.

### 3.3. ALPS-Index

**Figure 3:**
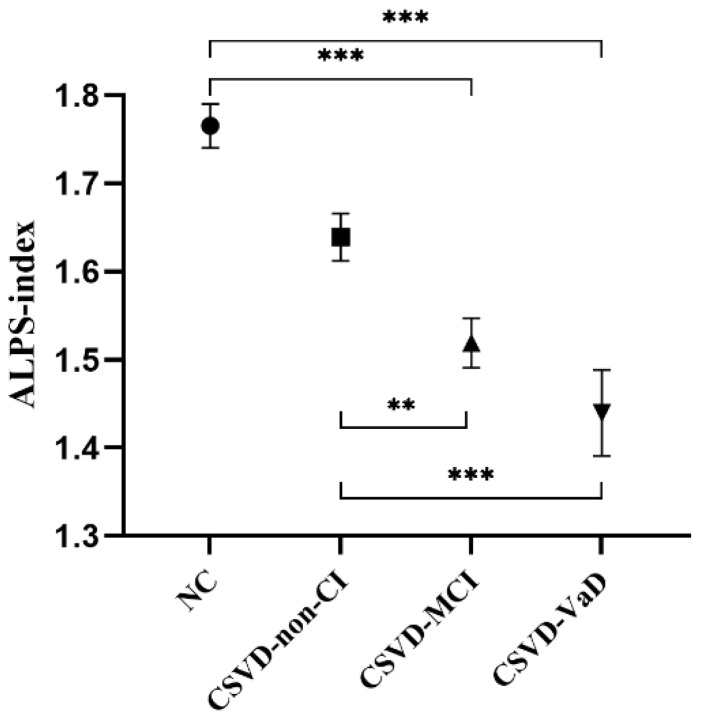
Comparison of the ALPS-index in the NC, CSVD-non-CI, CSVD-MCI, and CSVD-VaD groups. Abbreviations: ALPS-index, the index of Diffusion Tensor Image Analysis along the Perivascular Space; NC, normal control; CSVD, cerebral small vessel disease; CI, cognitive impairment; MCI, mild cognitive impairment; VaD, vascular dementia. Values are presented as mean and standard error of mean. ●: Mean ALPS-index in the NC group, ■: Mean ALPS-index in the CSVD-non-CI group, ▲: Mean ALPS-index in the CSVD-MCI group, ▼: Mean ALPS-index in the CSVD-VaD group, **: *p* < 0.01, ***: *p* < 0.001.

GLM was applied to the ALPS-index analysis between the NC, CSVD-non-CI, CSVD-MCI, and CSVD-VaD groups, with gender, age, hypertension, and education years as covariates. Markable differences in the ALPS-index were observed between the NC group (1.76 ± 0.18), CSVD-non-CI group (1.64 ± 0.20), CSVD-MCI group (1.52 ± 0.20), and CSVD-VaD group (1.44 ± 0.26) (*p* < 0.001) (Table 3). In the post hoc analysis, the ALPS-index was markable different between the NC and CSVD-MCI groups (*p* < 0.001), NC and CSVD-VaD groups (*p* < 0.001), CSVD-non-CI and CSVD-MCI groups (*p* = 0.003), and CSVD-non-CI and CSVD-VaD groups (*p* < 0.001) after Bonferroni corrections. However, no markable differences in the ALPS-index were found between the NC and CSVD-non-CI groups (*p* = 0.328) or the CSVD-MCI and CSVD-VaD groups (*p* = 0.469) (Figure 3).

### 3.4. Correlation Analysis between Demographics and ALPS-Index

**Figure 4:**
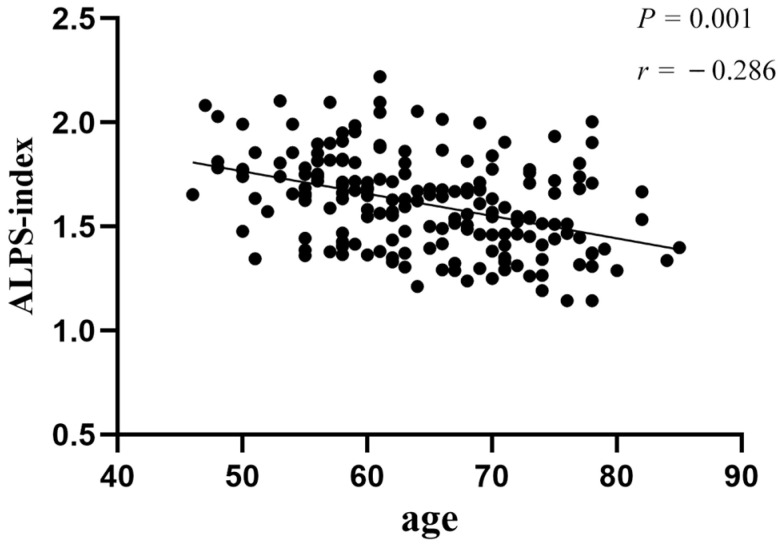
Correlation between ALPS-index and age in all CSVD patients. Abbreviations: ALPS-index, the index of Diffusion Tensor Image Analysis along the Perivascular Space; CSVD, cerebral small vessel disease.

Pearson correlation analysis revealed that age was negatively associated with the ALPS-index in the CSVD-non-CI (r = −0.373, *p* = 0.005) and CSVD-VaD (r = −0.409, *p* = 0.027) groups. The correlation coefficient between age and ALPS-index in all CSVD patients was −0.286 (*p* < 0.001) (Figure 4). There was no remarkable relationship between age and ALPS-index in the NC (*p* = 0.224) and CSVD-MCI (*p* = 0.462) groups. Furthermore, no correlations were found between the ALPS-index and other demographic data.

### 3.5. Correlation Analysis between ALPS-Index and CSVD Neuroimaging Features

**Figure 5:**
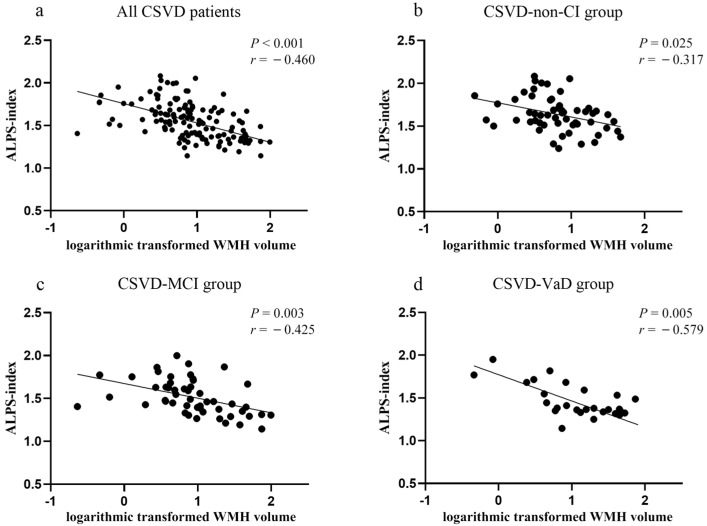
Correlation between ALPS-index and WMH volume in CSVD patients. (**a**) correlation between ALPS-index and WMH volume in all CSVD patients, (**b**) correlation between ALPS-index and WMH volume in CSVD-non-CI group, (**c**) correlation between ALPS-index and WMH volume in CSVD-MCI group, (**d**) correlation between ALPS-index and WMH volume in CSVD-VaD group. Abbreviations: ALPS-index, the index of Diffusion Tensor Image Analysis along the Perivascular Space; NC, normal control; CSVD, cerebral small vessel disease; CI, cognitive impairment; MCI, mild cognitive impairment; VaD, vascular dementia; WMH, white matter hyperintensities.

A negative association between white matter volume and ALPS-index was found in the NC group (r = −0.311, *p* = 0.032), but not in the CSVD-non-CI, CSVD-MCI, or CSVD-VaD groups. A significant negative correlation between the WMH volume and ALPS-index was indicated in all CSVD patients (r = −0.460, *p* < 0.001) (CSVD-non-CI group: r = −0.317, *p* = 0.025; CSVD-MCI group: r = −0.425, *p* = 0.003; CSVD-VaD group: r = −0.579, *p* = 0.005; Figure 5). The number of CMBs was negatively correlated with the ALPS-index in the CSVD-non-CI group (r = −0.404, *p* = 0.004). There was no correlation between the number of LIs and presence of EPVS-bg with the ALPS-index in each group.

### 3.6. Relationship between ALPS-Index and Cognitive Performance

**Figure 6:**
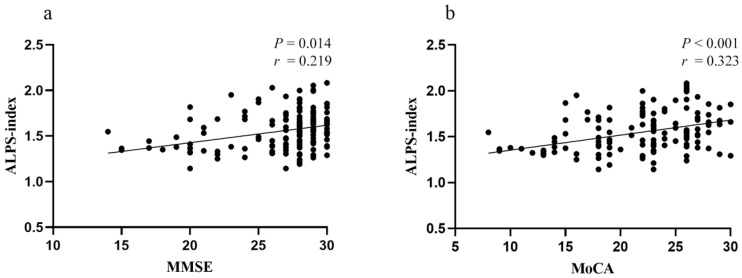
Correlation between ALPS-index and general cognitive function in all CSVD patients. (**a**) Positive correlation between ALPS-index and MMSE scores. (**b**) Positive correlation between ALPS-index and MoCA scores. Abbreviations: ALPS-index, the index of Diffusion Tensor Image Analysis along the Perivascular Space; CSVD, cerebral small vessel disease; MMSE, Mini-Mental State Examination; MoCA, Montreal Cognitive Assessment.

Partial correlation analysis was applied in order to determine the relationship between ALPS-index and cognitive performance with gender, age, education years, hypertension, WMH volume, and number of CMBs as covariates. The ALPS-index was found to be positively correlated with MMSE (r = 0.219, *p* = 0.014) and MoCA (r = 0.323, *p* < 0.001) scores in all CSVD patients (Figure 6). In addition, episodic memory scores were positively related to the ALPS-index in the CSVD-MCI group (r = 0.340, *p* = 0.024). No other significant relationships between ALPS-index and cognitive domains were found between the NC, CSVD-non-CI, CSVD-MCI, and CSVD-VaD groups.

### 3.7. Mediation Analysis

**Figure 7:**
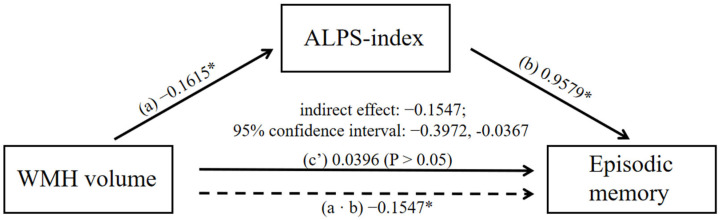
Mediating effect among WMH volume, ALPS-index, and episodic memory score in the CSVD-MCI group. Solid lines indicate direct effects (c’); dashed lines indicate indirect effects (a ∙ b). Associations are given as non-standardized regression coefficients (non-standardized ß). Gender, age, hypertension, and years of education were used as covariates. Abbreviations: WMH, white matter hyperintensities; ALPS-index, the index of Diffusion Tensor Image Analysis along the Perivascular Space; CSVD, cerebral small vessel disease; MCI, mild cognitive impairment. *: *p* < 0.05.

In order to further examine whether the ALPS-index could fully or partially associate WMH and CI, a mediation model was structured between the ALPS-index, WMH volumes, and cognition. It was found that the ALPS-index significantly mediated the relationship between WMH volumes and episodic memory (indirect effect: −0.1547; 95% confidence interval: −0.3972, −0.0367; Figure 7). With the exception of significant mediation on memory in the CSVD-MCI group, no other significant mediations were found.

## 4. Discussion

Our study reported, for the first time, that (1) the ALPS-index was a sensitive indicator that could distinguish MCI in CSVD patients; (2) the ALPS-index independently influenced episodic memory in CSVD patients with MCI; and (3) the ALPS-index mediated the relationship between WMH and episodic memory in CSVD patients with MCI. The relationship between ALPS-index and cognition was only found in MCI, highlighting the reliability of the ALPS-index in the early recognition of CI in CSVD patients. 

Our results suggested that the ALPS-index might be a sensitive parameter for early recognition of cognitive deficits, and showed a decreasing trend with the occurrence and development of the CSVD. Previously, the ALPS-index has also shown good performance in distinguishing cognitive decline in CSVD [23]. Similarly, the ALPS-index was also revealed to be relative to CSVD burden in cerebral amyloid angiopathy [24]. Notably, compared with these previous studies, we have applied a more detailed grouping of cognition, and thus, our results reflect the sensitive recognition ability of ALPS-index in detecting early cognitive decline in CSVD. However, there were no markable differences between the CSVD-MCI and CSVD-VaD groups, which was consistent with previous studies, demonstrating that the ALPS-index was similar between MCI and dementia in PD and AD patients [15,16,25]. However, since our sample size was relatively small, more studies are needed to further examine the function of the ALPS-index in the classification of CI.

In order to further explore the role of the ALPS-index in the CI of CSVD patients, correlation analyses were conducted to (1) determine the association between ALPS-index and specific cognitive parameters, and (2) identify ALPS-index-related CSVD neuroimaging features and demographic indicators. The ALPS-index was found to be independently correlated to global cognitive function in subjects with CSVD, adjusted for gender, hypertension, age, years of education, WMH volume, and CMB number. Interestingly, although the GS is believed to have a role in cognitive function, no strong association between the ALPS-index and general cognitive function has been reported [16,25]. Thus, we next examined the association between each cognitive domain and ALPS-index in each group of subjects in our study. A moderate independent positive correlation between ALPS-index and episodic memory was only found in CSVD patients with MCI. The earliest cognitive manifestation in CSVD is reportedly executive function [26]. However, no significant relationship between executive function and ALPS-index was found in our study. Similarly, in the study conducted by Jie Tang et al., memory was also found to be correlated with ALPS-index in CSVD patients [23]. The ROI of the ALPS-index in this study was located on the left side of the lateral ventricle, which is intersected by the superior longitudinal fasciculus and corticospinal system. Studies have shown that the impairment of superior longitudinal fasciculus in CSVD was related to cognitive decline [22,27]. The superior longitudinal fasciculus is the association fiber that interlinks the frontal, occipital, parietal, and temporal lobes. Brain regions connected by the left superior longitudinal fasciculus, such as the prefrontal cortex, angular gyrus, and superior marginal gyrus, are responsible for encoding and governing episodic memory formation [28,29].

Age and WMH volume were also found to be negatively related to the ALPS-index in CSVD patients. WMH is a critical neuroimaging feature in CSVD. Several vascular risk factors have been revealed to be involved in the decline of the ALPS-index in the elderly, such as aging, apnea-hypopnea index, hypertension, and diabetes [9,30,31]. Coincidentally, these factors are all closely associated with WMH [32].

Based on these correlation analyses, we then performed mediation analysis between WMH volume, ALPS-index, and cognitive function. Our data suggested that the ALPS-index only mediated the relationship between WMH and episodic memory only in CSVD patients with MCI. To our knowledge, this is the first time that this type of mediating relationship has been described. A lack of oxygen, energy, and nutrients, which can be caused by pathological alterations in the cerebral small vessels in CSVD, contributes to damage to the blood–brain barrier by oxidative stress and inflammation, in which WMH is a typical and sensitive manifestation [33]. Oxidative stress and inflammation lead to the production of metabolic waste, which, when deposited in the tissue, causes damage and impairs cerebral function. The GS plays a role in the elimination of waste products and is partly involved in the redistribution of substances required for metabolism, which compensates for the decline in cerebral function [10]. Since the ALPS-index reflects the speed of glymphatic circulation, a positive correlation between ALPS-index and cognition was found. In a study of PD, the ALPS-index was also found to be negatively correlated with oxidative stress status [25]. When calculating the ALPS-index, the influence of WMH on average diffusivity was eliminated as a denominator in the formula, which may explain why we observed a negative relationship between WMH and ALPS-index.

It is worth noting that PVSs are important transport tunnels in the GS. The expression and polarization of AQP4 on the endfeet of astrocytes abutting the PVS is a strong regulator of normal glymphatic function [10,34]. EPVS is a typical neuroimaging feature in CSVD, and AQP4 polarity disorder has also been reported in CSVD [3,5,35]. In addition, WMH was found to be correlated with EPVS in CSVD and obstructive sleep apnea [36,37]. EPVSs have also been associated with a high risk of dementia [34]. However, few studies have identified a direct correlation between the quantitative indicators of EPVS and cognitive parameters [38,39]. This might be because previous studies focused on the number and volume of EPVSs and ignored the function of the GS. In acute ischemic stroke, glymphatic inflow was accelerated by spreading depolarization together with subsequent vasoconstriction, which resulted in CSF entering the tissue along the perivascular flow channels and, in turn, enlarging the PVSs [40]. EPVS may, therefore, be the result of GS compensation in CSVD. Thus, the ALPS-index provides a novel approach for the study of EPVS in CSVD. Notably, no significant correlation between WMH volume and episodic memory were observed in CSVD patients with MCI. The effect of WMH on early cognitive decline is controversial, and both positive and negative correlations have been reported [41,42,43]. The effect and mechanism of WMH on cognition needs to be studied further.

## 5. Limitations

There were several limitations in this study. First, based on previous studies, we only considered the left ALPS-index and not the right ALPS-index in our study. Second, the existence of subjective cognitive decline, which might influence neuroimaging parameters, was not evaluated in subjects without CI. Third, the sample size in this study was limited. Fourth, the age among groups was not completely matched, and some participants in the NC group also had vascular risk factors, which might have partly affected the research results. Last but not least, we did not explore the relationship between ALPS-index and cortical neuroimaging features of CSVD, such as cortical atrophy, which is an emerging feature of CSVD that is usually progressive and documented mainly in patients with acute stroke of the lacunar type [44].

In the future, we will focus on furthering our understanding on the internal mechanism of the ALPS-index on cognition, and examine the relationship between glymphatic dysfunction and cortical neuroimaging features.

## 6. Conclusions

The ALPS-index showed good performance in early recognition of CI, and glymphatic dysfunction mediated the relationship between WMH and CI in CSVD in our study. The GS might be involved in the preservation of cognitive function and compensate for cerebral functional deficits caused by CSVD. These findings may provide new evidence for the influence of ALPS on cognitive decline in CSVD.

## Figures and Tables

**Table 1:** Demographics of the participants enrolled in this study.

Variables	NC Group (*n* = 52)	CSVS-Non-CI Group (*n* = 56)	CSVD-MCI Group (*n* = 52)	CSVD-VaD Group (*n* = 29)	F\χ^2^	*p* Value
Gender (F/M)	25/27	28/28	26/26	15/14	0.106	0.991
Age (years, mean ± s.d.)	58.88 ± 6.41	66.28 ± 9.25	67.48 ± 6.74	66.07 ± 8.75	12.87	<0.001 ^a–c^
Education (years, mean ± s.d.)	14.48 ± 3.58	12.96 ± 3.46	10.19 ± 3.07	9.41 ± 3.19	21.75	<0.001 ^b–e^
**Vascular risk factors**						
Hypertension, *n* (%)	24 (46.2%)	40 (71.4%)	39 (75.0%)	19 (65.5%)	13.203	0.004 ^a–c^
Diabetes mellitus, *n* (%)	8 (15.4%)	12 (21.4%)	16 (31.4%)	6 (20.7%)	3.745	0.298
Hyperlipidemia, *n* (%)	13 (25.0%)	12 (21.4%)	8 (15.7%)	2 (6.9%)	1.36	0.253
Smoking, *n* (%)	10 (19.2%)	10 (17.9%)	12 (23.5%)	5 (17.2%)	0.491	0.920
Alcohol drinking, *n* (%)	10 (19.2%)	9 (16.7%)	11 (21.6%)	1 (3.4%)	4.41	0.214

Fisher’s exact test was used to determine the association between hyperlipidemia and alcohol drinking. A Chi-square test was used to analyze the association between gender, hypertension, diabetes mellitus, and smoking. A one-way analysis of variance (ANOVA) was used to analyze other variables. Abbreviations: NC, normal control; CSVD, cerebral small vessel disease; CI, cognitive impairment; MCI, mild cognitive impairment; VaD, vascular dementia; F, female; M, male; s.d., standard deviation. ^a^ remarkable difference between NC group and CSVD-non-CI group, ^b^ remarkable difference between NC group and CSVD-MCI group, ^c^ remarkable difference between NC group and CSVD-VaD group, ^d^ remarkable difference between CSVD-non-CI group and CSVD-MCI group, ^e^ remarkable difference between CSVD-non-CI group and CSVD-VaD group.

**Table 2:** Cognitive function in NC, CSVD-non-CI, CSVD-MCI, and CSVD-VaD groups.

Variables	NC Group (*n* = 52)	CSVS-Non-CI Group (*n* = 56)	CSVD-MCI Group (*n* = 52)	CSVD-VaD Group (*n* = 29)	Statistics	*p* Value
**General cognitive** **function**						
MMSE	29.36 ± 0.77	28.84 ± 1.06	27.48 ± 1.39	20.24 ± 2.73	277.736	<0.001 ^b–f^
MoCA	27.40 ± 1.30	26.87 ± 1.41	21.07 ± 2.60	14.52 ± 3.42	289.684	<0.001 ^b–f^
**Cognitive** **domains**						
episodic memory	0.34 ± 0.09	0.44 ± 0.08	−0.23 ± 0.08	−1.03 ± 0.11	38.164	<0.001 ^b–f^
Z-linguistic function	0.53 ± 0.10	0.30 ± 0.10	−0.31 ± 0.10	−1.21 ± 0.15	30.603	<0.001 ^b–f^
Z-processing speed	0.40 ± 0.10	0.32 ± 0.09	−0.38 ± 0.09	−0.88 ± 0.14	24.675	<0.001 ^b–f^
Z-executive function	0.17 ± 0.11	0.27 ± 0.09	−0.26 ± 0.10	−0.53 ± 0.15	8.724	<0.05 ^b–e^
Z-visuospatial	0.30 ± 0.10	0.34 ± 0.09	−0.06 ± 0.10	−1.54 ± 0.15	37.914	<0.01 ^c–f^

All variables are shown as mean ± standard deviation. A general linear modal (GLM) was used to compare cognitive function, adjusted for gender, age, hypertension, and education years. Abbreviations: NC, normal control; CSVD, cerebral small vessel disease; CI, cognitive impairment; MCI, mild cognitive impairment; VaD, vascular dementia; MMSE, Mini-Mental State Examination; MoCA, Montreal Cognitive Assessment. ^b^ remarkable difference between NC group and CSVD-MCI group, ^c^ remarkable difference between NC group and CSVD-VaD group, ^d^ remarkable difference between CSVD-non-CI group and CSVD-MCI group, ^e^ remarkable difference between CSVD-non-CI group and CSVD-VaD group, ^f^ remarkable difference between CSVD-MCI group and CSVD-VaD group.

**Table 3:** ALPS-index and neuroimaging features in the NC, CSVD-non-CI, CSVD-MCI, and CSVD-VaD groups.

Variables	NC Group (*n* = 52)	CSVS-Non-CI Group (*n* = 56)	CSVD-MCI Group (*n* = 52)	CSVD-VaD Group (*n* = 29)	Statistics	*p* Value
ALPS-index (mean ± s. d.)	1.76 ± 0.18	1.64 ± 0.20	1.52 ± 0.20	1.44 ± 0.26	13.706	<0.001 ^b–e^
**neuroimaging features**						
WMH volume (mL, median (interquartile range))	0.93 (0.87)	6.65 (8.84)	8.00 (15.69)	13.14 (34.93)	95.429	<0.001 ^a–c,e^
CMB numbers median (interquartile range)	0	0 (2.00)	0 (5.00)	2.00 (28.00)	56.604	<0.001 ^a–c,e,f^
LI numbers median (interquartile range)	0	0 (1.00)	1.00 (2.00)	1.00 (2.00)	54.170	<0.05 ^a–e^
EPVS-bg, *n* (%)	0 (0%)	19 (33.9%)	19 (36.5%)	11 (37.9%)	25.293	<0.001 ^a–c^
global gray matter volume (mL, mean ± s.d.)	547.52 ± 46.58	546.67 ± 50.30	539.32 ± 51.20	543.96 ± 57.33	1.103	0.349
global white matter volume (mL, mean ± s.d.)	463.36 ± 60.50	466.46 ± 57.20	448.13 ± 52.42	449.37 ± 69.49	0.514	0.673
cerebrospinal fluid volume (mL, mean ± s.d.)	309.21 ± 37.08	337.38 ± 42.64	334.80 ± 56.48	341.77 ± 60.29	5.246	0.001 ^c^

The Kruskal–Wallis test was used to analyze WMH volume, CMB numbers, and LI numbers. The Chi-square test was used to analyze EPVS-bg. A general linear modal (GLM) was used to analyze other variables, adjusted for gender, age, hypertension, and education years. Abbreviations: ALPS-index, the index of Diffusion Tensor Image Analysis along the Perivascular Space; NC, normal control; CSVD, cerebral small vessel disease; CI, cognitive impairment; MCI, mild cognitive impairment; VaD, vascular dementia; WMH, white matter hyperintensities; CMB, cerebral microbleed; LI, lacunar infarction; EPVS-bg, enlarged perivascular space on basal ganglia; mL, milliliter; s.d., standard deviation. ^a^ remarkable difference between NC group and CSVD-non-CI group, ^b^ remarkable difference between NC group and CSVD-MCI group, ^c^ remarkable difference between NC group and CSVD-VaD group, ^d^ remarkable difference between CSVD-non-CI group and CSVD-MCI group, ^e^ remarkable difference between CSVD-non-CI group and CSVD-VaD group, ^f^ remarkable difference between CSVD-MCI group and CSVD-VaD group.

## Data Availability

If anyone is interested in extrapolating their data for further validation, the MRI images can be made available to the scientific community from the corresponding author.

## References

[1] Rudilosso S., Rodriguez-Vazquez A., Urra X., Arboix A. (2022). The Potential Impact of Neuroimaging and Translational Research on the Clinical Management of Lacunar Stroke. Int. J. Mol. Sci..

[2] Wardlaw J.M., Smith C., Dichgans M. (2019). Small vessel disease: Mechanisms and clinical implications. Lancet Neurol..

[3] Cannistraro R.J., Badi M., Eidelman B.H., Dickson D.W., Middlebrooks E.H., Meschia J.F. (2019). CNS small vessel disease A clinical review. Neurology.

[4] Kasper S., Bancher C., Eckert A., Forstl H., Frolich L., Hort J., Korczyn A.D., Kressig R.W., Levin O., Palomo M.S.M. (2020). Management of mild cognitive impairment (MCI): The need for national and international guidelines. World J. Biol. Psychiatry.

[5] Iliff J.J., Wang M.H., Liao Y.H., Plogg B.A., Peng W.G., Gundersen G.A., Benveniste H., Vates G.E., Deane R., Goldman S.A. (2012). A Paravascular Pathway Facilitates CSF Flow Through the Brain Parenchyma and the Clearance of Interstitial Solutes, Including Amyloid beta. Sci. Transl. Med..

[6] Plog B.A., Nedergaard M., Abbas A.K., Aster J.C. (2018). The Glymphatic System in Central Nervous System Health and Disease: Past, Present, and Future. Annual Review of Pathology: Mechanisms of Disease.

[7] Nedergaard M., Goldman S.A. (2020). Glymphatic failure as a final common pathway to dementia. Science.

[8] Mestre H., Kostrikov S., Mehta R.I., Nedergaard M. (2017). Perivascular spaces, glymphatic dysfunction, and small vessel disease. Clin. Sci..

[9] Zhang Y., Zhang R.T., Ye Y.Q., Wang S.Y., Jiaerken Y., Hong H., Li K.C., Zeng Q.Z., Luo X., Xu X.P. (2021). The Influence of Demographics and Vascular Risk Factors on Glymphatic Function Measured by Diffusion Along Perivascular Space. Front. Aging Neurosci..

[10] Mestre H., Mori Y., Nedergaard M. (2020). The Brain’s Glymphatic System: Current Controversies. Trends Neurosci..

[11] Iliff J.J., Wang M.H., Zeppenfeld D.M., Venkataraman A., Plog B.A., Liao Y.H., Deane R., Nedergaard M. (2013). Cerebral Arterial Pulsation Drives Paravascular CSF-Interstitial Fluid Exchange in the Murine Brain. J. Neurosci..

[12] Ringstad G., Vatnehol S.A.S., Eide P.K. (2017). Glymphatic MRI in idiopathic normal pressure hydrocephalus. Brain.

[13] Taoka T., Masutani Y., Kawai H., Nakane T., Matsuoka K., Yasuno F., Kishimoto T., Naganawa S. (2017). Evaluation of glymphatic system activity with the diffusion MR technique: Diffusion tensor image analysis along the perivascular space (DTI-ALPS) in Alzheimer’s disease cases. Jpn. J. Radiol..

[14] Zhang W.H., Zhou Y., Wang J.A., Gong X.X., Chen Z.C., Zhang X.T., Cai J.S., Chen S.Y., Fang L., Sun J.Z. (2021). Glymphatic clearance function in patients with cerebral small vessel disease. Neuroimage.

[15] Ma X.X., Li S.H., Li C.M., Wang R., Chen M., Chen H.B., Su W. (2021). Diffusion Tensor Imaging Along the Perivascular Space Index in Different Stages of Parkinson’s Disease. Front. Aging Neurosci..

[16] Steward C.E., Venkatraman V.K., Lui E., Malpas C.B., Ellis K.A., Cyarto E.V., Vivash L., O’Brien T.J., Velakoulis D., Ames D. (2021). Assessment of the DTI-ALPS Parameter Along the Perivascular Space in Older Adults at Risk of Dementia. J. Neuroimaging.

[17] Pantoni L. (2010). Cerebral small vessel disease: From pathogenesis and clinical characteristics to therapeutic challenges. Lancet Neurol..

[18] Katzman R., Zhang M.Y., Ouang Ya Q., Wang Z.Y., Liu W.T., Yu E., Wong S.C., Salmon D.P., Grant I. (1988). A Chinese version of the Mini-Mental State Examination; impact of illiteracy in a Shanghai dementia survey. J. Clin. Epidemiol..

[19] Lu J., Li D., Li F., Zhou A.H., Wang F., Zuo X.M., Jia X.F., Song H.Q., Jia J.P. (2011). Montreal Cognitive Assessment in Detecting Cognitive Impairment in Chinese Elderly Individuals: A Population-Based Study. J. Geriatr. Psychiatry Neurol..

[20] Gorelick P.B., Scuteri A., Black S.E., DeCarli C., Greenberg S.M., Iadecola C., Launer L.J., Laurent S., Lopez O.L., Nyenhuis D. (2011). Vascular Contributions to Cognitive Impairment and Dementia A Statement for Healthcare Professionals From the American Heart Association/American Stroke Association. Stroke.

[21] Schmidt P., Gaser C., Arsic M., Buck D., Forschler A., Berthele A., Hoshi M., Ilg R., Schmid V.J., Zimmer C. (2012). An automated tool for detection of FLAIR-hyperintense white-matter lesions in Multiple Sclerosis. Neuroimage.

[22] Huang L.L., Chen X., Sun W.S., Chen H.F., Ye Q., Yang D., Li M.C., Luo C.M., Ma J.Y., Shao P.F. (2021). Early Segmental White Matter Fascicle Microstructural Damage Predicts the Corresponding Cognitive Domain Impairment in Cerebral Small Vessel Disease Patients by Automated Fiber Quantification. Front. Aging Neurosci..

[23] Tang J., Zhang M., Liu N., Xue Y., Ren X., Huang Q., Shi L., Fu J. (2022). The Association Between Glymphatic System Dysfunction and Cognitive Impairment in Cerebral Small Vessel Disease. Front. Aging Neurosci..

[24] Xu J., Su Y., Fu J., Wang X., Nguchu B.A., Qiu B., Dong Q., Cheng X. (2022). Glymphatic dysfunction correlates with severity of small vessel disease and cognitive impairment in cerebral amyloid angiopathy. Eur. J. Neurol..

[25] Chen H.L., Chen P.C., Lu C.H., Tsai N.W., Yu C.C., Chou K.H., Lai Y.R., Taoka T., Lin W.C. (2021). Associations among Cognitive Functions, Plasma DNA, and Diffusion Tensor Image along the Perivascular Space (DTI-ALPS) in Patients with Parkinson’s Disease. Oxidative Med. Cell. Longev..

[26] Hamilton O.K.L., Backhouse E.V., Janssen E., Jochems A.C.C., Maher C., Ritakari T.E., Stevenson A.J., Xia L.H., Deary I.J., Wardlaw J.M. (2021). Cognitive impairment in sporadic cerebral small vessel disease: A systematic review and meta-analysis. Alzheimer’s Dement..

[27] Hu A.M., Ma Y.L., Li Y.X., Han Z.Z., Yan N., Zhang Y.M. (2022). Association between Changes in White Matter Microstructure and Cognitive Impairment in White Matter Lesions. Brain Sci..

[28] Rubinstein D.Y., Camarillo-Rodriguez L., Serruya M.D., Herweg N.A., Waldman Z.J., Wanda P.A., Sharan A.D., Weiss S.A., Sperling M.R. (2021). Contribution of left supramarginal and angular gyri to episodic memory encoding: An intracranial EEG study. Neuroimage.

[29] Orth M., Wagnon C., Neumann-Dunayevska E., Kaller C.P., Kloppel S., Meier B., Henke K., Peter J. (2022). The left prefrontal cortex determines relevance at encoding and governs episodic memory formation. Cereb. Cortex.

[30] Siow T.Y., Toh C.H., Hsu J.L., Liu G.H., Lee S.H., Chen N.H., Fu C.J., Castillo M., Fang J.T. (2022). Association of Sleep, Neuropsychological Performance, and Gray Matter Volume With Glymphatic Function in Community-Dwelling Older Adults. Neurology.

[31] Benveniste H., Nedergaard M. (2022). Cerebral small vessel disease: A glymphopathy?. Curr. Opin. Neurobiol..

[32] Lockhart S.N., Schaich C.L., Craft S., Sachs B.C., Rapp S.R., Jung Y., Whitlow C.T., Sai K.K.S., Cleveland M., Williams B.J. (2022). Associations among vascular risk factors, neuroimaging biomarkers, and cognition: Preliminary analyses from the Multi-Ethnic Study of Atherosclerosis (MESA). Alzheimer’s Dement..

[33] Wong S.M., Jansen J.F.A., Zhang C.E., Hoff E.I., Staals J., van Oostenbrugge R.J., Backes W.H. (2019). Blood-brain barrier impairment and hypoperfusion are linked in cerebral small vessel disease. Neurology.

[34] Xu Z.H., Li F.F., Xing D.X., Song H.Y., Chen J.S., Duan Y., Yang B.Q. (2021). A Novel Imaging Biomarker for Cerebral Small Vessel Disease Associated with Cognitive Impairment: The Deep-Medullary-Veins Score. Front. Aging Neurosci..

[35] Liu X.L., Ouyang F.B., Hu L.T., Sun P., Yang J., Sun Y.J., Liao M.S., Lan L.F., Pei Z., Fan Y.H. (2022). Mesenchymal Stem Cells Improve Cognitive Impairment and Reduce A beta Deposition via Promoting AQP4 Polarity and Relieving Neuroinflammation in Rats with Chronic Hypertension-Induced Cerebral Small-Vessel Disease. Front. Aging Neurosci..

[36] Jia Y.L., Liu C.L., Li H., Li X.N., Wu J., Zhao Y.M., Xu M.Y., Yu H.T., Guan Z.T., Sun S.N. (2021). Enlarged Perivascular Space and Its Correlation with Polysomnography Indicators of Obstructive Sleep Apnea. Nat. Sci. Sleep.

[37] Shen M., Wei G.R., Cheng M., Jiang H. (2020). Association between Enlarged Perivascular Spaces and Internal Carotid Artery Stenosis: A Study in Patients Diagnosed by Digital Subtraction Angiography. J. Stroke Cerebrovasc. Dis..

[38] Gertje E.C., van Westen D., Panizo C., Mattsson-Carlgren N., Hansson O. (2021). Association of Enlarged Perivascular Spaces and Measures of Small Vessel and Alzheimer Disease. Neurology.

[39] Gyanwali B., Vrooman H., Venketasubramanian N., Wong T.Y., Cheng C.Y., Chen C., Hilal S. (2019). Cerebral Small Vessel Disease and Enlarged Perivascular Spaces-Data from Memory Clinic and Population-Based Settings. Front. Neurol..

[40] Mestre H., Du T., Sweeney A.M., Liu G.J., Samson A.J., Peng W.G., Mortensen K.N., Staeger F.F., Bork P.A.R., Bashford L. (2020). Cerebrospinal fluid influx drives acute ischemic tissue swelling. Science.

[41] Alber J., Alladi S., Bae H.-J., Barton D.A., Beckett L.A., Bell J.M., Berman S.E., Biessels G.J., Black S.E., Bos I. (2019). White matter hyperintensities in vascular contributions to cognitive impairment and dementia (VCID): Knowledge gaps and opportunities. Alzheimer’s Dement..

[42] Clancy U., Gilmartin D., Jochems A.C.C., Knox L., Doubal F.N., Wardlaw J.M. (2021). Neuropsychiatric symptoms associated with cerebral small vessel disease: A systematic review and meta-analysis. Lancet Psychiatry.

[43] Dao E., Tam R., Hsiung G.Y.R., ten Brinke L., Crockett R., Barha C.K., Yoo Y., Al Keridy W., Doherty S.H., Laule C. (2021). Exploring the Contribution of Myelin Content in Normal Appearing White Matter to Cognitive Outcomes in Cerebral Small Vessel Disease. J. Alzheimer’s Dis..

[44] Grau-Olivares M., Arboix A., Junque C., Arenaza-Urquijo E.M., Rovira M., Bartres-Faz D. (2010). Progressive gray matter atrophy in lacunar patients with vascular mild cognitive impairment. Cerebrovasc. Dis..

